# Revisiting Lebedev’s one-century old experiment

**DOI:** 10.1038/s41598-022-17398-3

**Published:** 2022-07-31

**Authors:** Shangcong Cheng

**Affiliations:** Molecular Foundry of Lawrence Berkeley National Laboratory, Berkeley, CA 94720 USA

**Keywords:** Materials science, Physics

## Abstract

One hundred years ago, world-famous scientist A. A. Lebedev performed a set of classical measurements on annealed optic crown glasses. He found that these glasses exhibited characteristic endothermic effects in a particular temperature range. To explain these phenomena, Lebedev proposed a hypothesis that the glasses contain tiny quartz crystals. This initial hypothesis was quickly disapproved, and the origin of the endothermic effect of glasses remains an unsolved puzzle. This work uses recently proposed nanoflake model of silica glass structure to explain the endothermic effect of various glasses. The new model differs from the popular continuous random network theory in that it emphasizes the medium-range ordering structure of glasses. According to the nanoflake based theory, the endothermic effect of glasses is caused by the transition from ordered one-dimensional structures into disordered structure in glasses. The new theory also predicts that the temperature range of the endothermic effect is dependent on both glass composition and cooling rates during glass formation.

## Introduction

**Figure 1:**
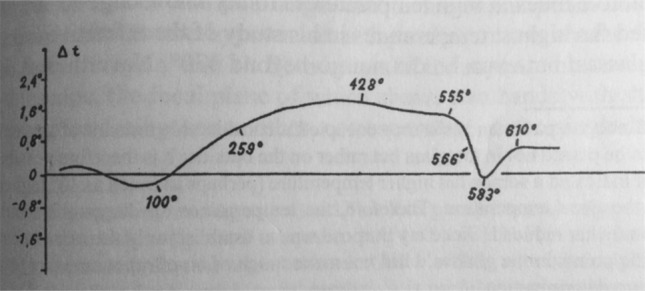
The experimental result of the temperature difference ∆t between the borosilicate crown glass and the neutral glass sample. The characteristic curve inflection between 555 and 610 °C shows a heat absorption process in the borosilicate crown glass. Reproduced from Ref.^1^.

In 1921, A. A. Lebedev published his classic paper, titled “The Polymorphism and Annealing of Glass”^1^. He investigated the heating and cooling curves of several glass samples up to 700 °C using the Roberts-Austen differential method. The glass specimen and the neutral control sample were placed side by side in an electric furnace; an Ag-constantan thermocouple measured the temperature difference ∆t between the glass sample and the neutral sample. Figure 1 is the experimental result for a borosilicate crown glass. The characteristic curve inflection in Fig. 1 shows that between 555 and 610 °C, a process in glass is accompanied by heat absorption. Lebedev also found that the crown glasses' refractive index and thermal expansion show a sharp change in the temperature range between 540 and 600 °C.

Understanding the unusual endothermic effect of optical glasses is essential to optical glass manufacture. It may also reveal the nature of glass state and glass transition. To interpret the experimental result, Lebedev proposed a hypothesis that correlates the observed heat absorption and other changes in glass physical properties with the modification transition from quartz crystal. This is based on the reasoning that the well-known $\alpha -\upbeta$ modification transition of quartz has a similar endothermic character within almost the same temperature region. However, Lebedev’s initial hypothesis that borosilicate crown glass contains an aggregate of highly dispersed quartz crystals was disproved by X-ray diffraction data, since no characteristic diffraction line of hexagonal crystal of $\upbeta$-quartz and trigonal crystal of $\alpha$-quartz could be found. A few years later, in 1924, A. Q. Tool and C. G. Echlin suggested that all the effects observed in the glass heating process may be explained by the transition of specific molecules, either simple or complex, or molecular arrangements^2^. In 1927, Lebedev published another paper, in which the original correlation of the endothermic effect with existing quartz crystals was replaced by correlation of the endothermic effect with glass modification transformation^3^. Based on the fundamental materials science principle that physical properties of materials must correlate with their internal structures, the hypothesis by Tool and Echlin or the modified Lebedev’s hypothesis are quite reasonable. And there is scientific motivation to search for the structural reason behind the endothermic effect. However, finding such a correlation has proved to be elusive. In 1995, it was even suggested that the problem of searching for the molecular arrangement and determining their sizes and shape corresponding to the endothermic effect is meaningless^4^. Although many studies and glass transition models were proposed, there is no commonly accepted structural explanation to this one-century-old experiment^5–9^.

This work shows that a newly proposed medium-range structure of glasses can explain the internal re-arrangement of borosilicate glasses that correlates with the endothermic effect^10,11^. In addition, this work will address how both cooling rate and chemical compensations can affect the temperature regions of the endothermic effect.

## Medium-range structure of silica glass

**Figure 2:**
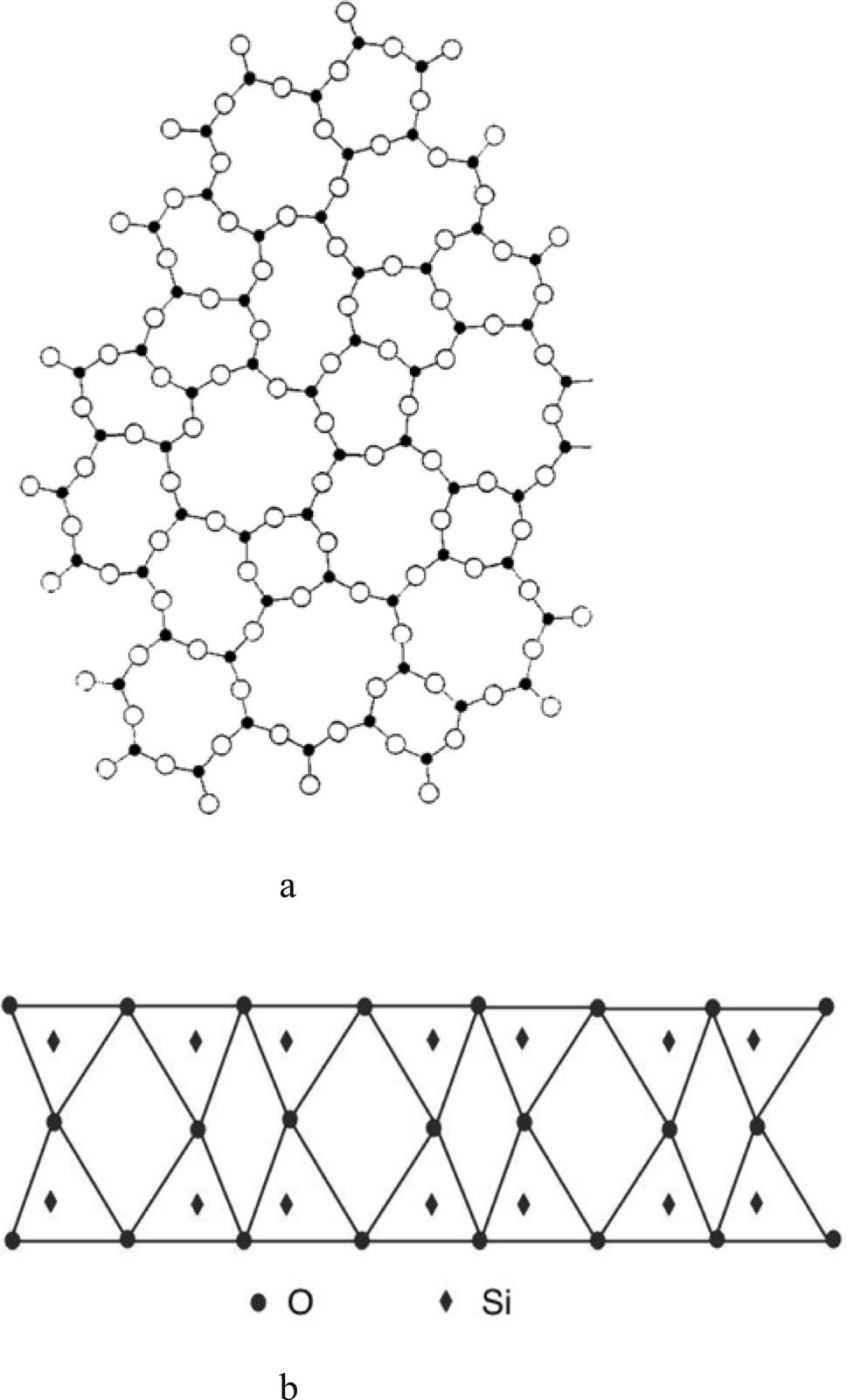
Recently proposed two-layer structure on facets of clusters in silica glass. (**a**) Top view from the direction perpendicular to the layers. (**b**) Side view of the layer structure. Reproduced from Ref.^10^.

From recent studies on the structure and properties of silica glass, the formation and evolution of medium-range ordering structure in silica glass have been revealed^11,12^. The core knowledge gained from these studies is the recognition of two different temperature regions in the glass transition process. The glass structures in these two temperature regions alter along different pathways, and all observed dynamic features need to be explained according to the structural rearrangements in the corresponding transition process. These two temperature regions for pure silica are separated by a critical temperature Tc of 1470 °C, which is the polymorphic inversion temperature between crystal $\upbeta$-cristobalite and $\upbeta$-tridymite. In the high-temperature region from the melting temperature Tm to the critical temperature Tc, SiO_4_ in the supercooled liquid forms embryonic clusters of $\upbeta$-cristobalite. These clusters may grow to form crystal nuclei and further to form larger crystal particles if the cooling rate is slow. However, if the cooling rate is fast, crystallization is avoided and these clusters enter a temperature zone lower than Tc before growing to form crystal nuclei. Because the clusters' formation pathway to $\upbeta$-cristobalite crystal is blocked in the lower temperature region, a one-dimension ordering structure on the clusters' facets (referred as nanoflake for convenience) is formed to minimize the system's free energy^12^. Figure 2a shows that the nanoflake consists of various membered-rings when viewed from the top, which are the same as described in Zachariasen’s continuous random network theory^13^; Fig. 2b shows the side view of nanoflake, which consists two layers of SiO_4_ tetrahedra with a thickness of about 0.8 nm, extended to about 2 nm in width^10^. (See supplemental Figs. 1 and 2).

As the temperature decreases further from Tc, more nanoflakes will form. The structural disorder to order transformation is continuous and has all the characteristics of a second-order phase transition in the Paul Ehrenfest classification scheme^11^. (See supplemental Figs. 3 and 4) The disorder to order transition has a characteristic ending temperature Tg and is an exothermic process. In contrast, in the reverse process of heating up, at a temperature lower than Tg, the heat energy is only spent on the increase of kinetic energy of molecules. But at Tg more heat energy has to be spent on overcoming the potential energy of the ordered structure. Thus, the glass absorbs more heat and shows the endothermic effect at Tg. Using this effect, Tg can practically be determined from the characteristic rise of the temperature dependence of the specific heat at Tg. An interesting feature of Tg is its cooling rate dependence. Rapidly quenched glass may have a higher Tg than well-annealed glass because some clusters in quenched glass may not have the chance of transforming to a more ordered structure. Furthermore, the cooling rate in the high-temperature range from melting temperature Tm to critical temperature Tc also influences Tg's value. The total number of formed clusters in a fast cooling is less than that in a slow cooling. Thus, the transformation process below Tc can be completed in a relatively narrow temperature range and with a higher Tg for more rapid cooling. Published data shows that Tg of silica glass is about 1200°C^14,15^, around which the endothermic effect of pure silica glass can be observed.

**Figure 3:**
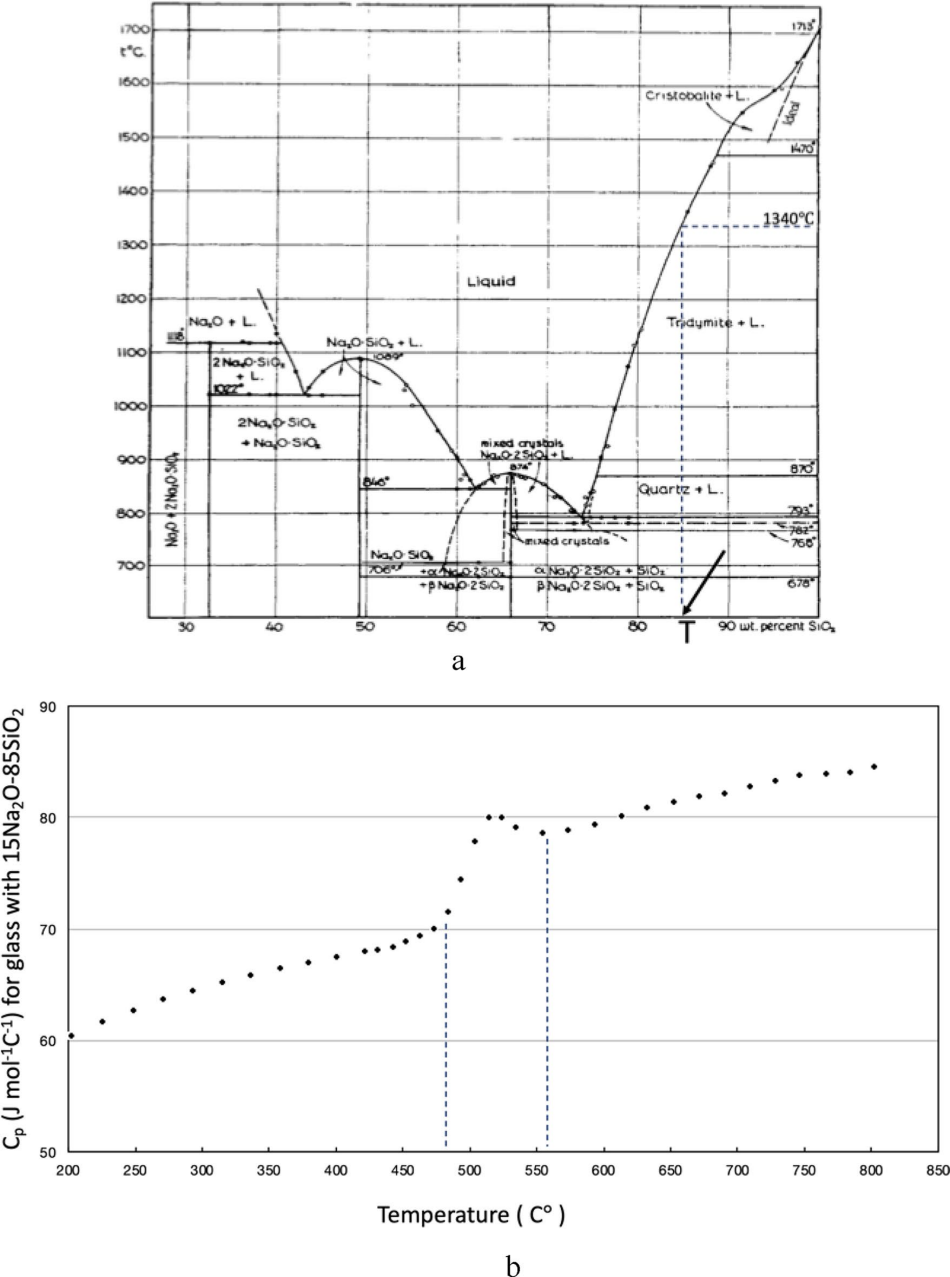
(**a**) The Na_2_O-SiO_2_ phase diagram. Point T, indicated by an arrow, represents sodium silicate glass with 15 wt.% of NaO_2_. For this particular glass, its liquidus temperature and critical temperature are found from the figure to be 1340 and 870 °C, respectively. Reproduced from Ref.^16^. (**b**) The heat capacity Cp of sodium silicate glass with 15 mol.% of NaO_2_ as a function of temperature. The data are taken from references^19,20^. The sharp rising of Cp between 480 and 560 °C in the figure indicates that heat absorption increases sharply in this temperature range.

**Figure 4:**
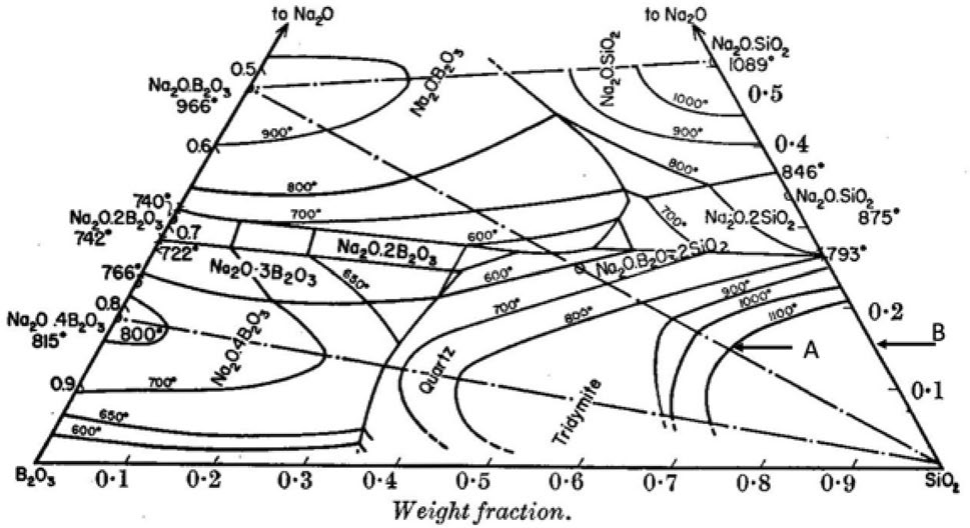
The phase diagram of the ternary system Na_2_O–B_2_O_3_–SiO_2_. Point A represents the glass with typical chemical compositions by weight % for borosilicate crown glass (15% Na_2_O, 15% B_2_O_3_, and 70% SiO_2_); point B represents the sodium silicate glass with 15 wt.% of NaO_2_. Glass A and other borosilicate crown glasses with slightly various compositions have different liquidus temperatures compared with glass B. All these liquids solidify to the same tridymite crystal. Reproduced from Ref.^21^.

## Endothermic effects of sodium silicate glasses

As Lebedev reported, the endothermic effect of borosilicate crown glasses is in the temperature region from 550 to 610 °C. The highest heating temperature in the Lebedev’s experiments is 700 °C. This is much lower than 1200 °C, where endothermic effect of silica glass can be observed. This is why Lebedev did not find the endothermic effect of pure silica in his experiments. The large difference in the temperature regions of endothermic effect between borosilicate crown glass and silica glass is caused by the difference in their chemical compositional. Sodium silicate glasses with various Na_2_O concentration can be used to illustrate the influence of chemical concentration on the temperature region of the endothermic effects.

Figure 3a is the Na_2_O-SiO_2_ phase diagram, which shows the variation of the liquidus temperature of the binary glass when the concentration of sodium oxide varies^16^. As sodium oxide in the glasses increases from 0 to 11.3 wt.%, the liquidus temperature of the glasses decreases from 1713 to 1470 °C. Because the corresponding solidification crystal of these glasses is $\upbeta$-cristobalite, which is the same as that of pure silica glass, the sodium cations are not involved in the entire process of crystal formation, including embryonic clusters formation. Sodium silicate glasses with less than 11.3 wt.% sodium oxide concentration have the same critical temperature Tc as pure silica glass at 1470 °C^17^. It is expected that the disorder to order transformation process below Tc takes a few hundred degrees of the temperature range to be completed. Thus, Tg of these glasses should be close to 1200 °C, same as pure silica glass; and the endothermic effect of all these glasses should be observed near 1200 °C.

Figure 3a also shows that the liquidus temperature for sodium silicate glasses in the composition range of 11.3–24.5 wt.% decreases from 1470 to 870 °C. The corresponding crystal in this temperature range through which the sodium silicate glass solidifies is $\upbeta$-tridymite, not $\upbeta$-cristobalite. The critical temperature Tc that separates the two different temperature regions is the polymorphic inversion temperature between crystal $\upbeta$-tridymite and $\upbeta$-quartz, which is 870 °C^17^. In the glass forming process above 870 °C, the formed clusters are $\upbeta$-tridymite embryos, not $\upbeta$-cristobalite embryos. Although the $\upbeta$-tridymite embryonic clusters have a different shape and number of facets than that of $\upbeta$-cristobalite, they still experience the disorder to order transformation in the temperature region lower than 870 °C. Because the arrangement of SiO_4_ tetrahedra in the base plan of $\upbeta$-tridymite hexagonal structure is the same as that in (111) plan of $\upbeta$-cristobalite face-centered cubic structure^18^, Figs. 2a and b can also be used to illustrate the stabilized structure of $\upbeta$-tridymite embryonic clusters formed on the facets. The ending temperature of this transition, Tg, is expected to be lower than 870 °C by a couple of hundred degrees and can be experimentally determined. For example, point T, indicated by an arrow in Fig. 3a, represents sodium silicate glass with 15 wt.% of NaO_2_. This particular glass's liquidus temperature and critical temperature are found from Fig. 3a to be 1340 and 870 °C, respectively. The temperature region of the endothermic effect of this glass can be determined from the experimental heat capacity data. The heat capacity Cp of sodium silicate glass with 15 mol.% of NaO_2_ as a function of temperature is found from references^19,20^ and is plotted in Fig. 3b. Since in the Na_2_O – SiO_2_ system, the compositions expressed in wt.% and in mol.% differ very little, the above found Cp data represents the thermal property of glass presented by point T in Fig. 3a. The sharp rising of Cp from 480 to 560 °C in Fig. 3b indicates that heat absorption increases rapidlyly within the temperature region from 480 to 560 °C. This temperature range of the sodium silicate glass, showing the endothermic effect, are very close to that observed for borosilicate crown glass by Lebedev, as shown in Fig. 1.

## Endothermic effects of borosilicate crown glass

Similar endothermic properties between sodium silicate glass with 15 wt.% of Na_2_O and Lebedev's borosilicate crown glasses can be explained by the phase diagram of the ternary system Na_2_O–B_2_O_3_–SiO_2_^21^. As shown in Fig. 4, point A represents glass with typical chemical compositions by weight % for borosilicate crown glass (15% Na_2_O, 15% B_2_O_3_, and 70% SiO_2_)^22^; point B represents sodium silicate glass with 15 wt.% of Na_2_O (discussed in the section above). Comparison of the two glass compositions shows that a small portion of SiO_2_ in glass B is replaced by B_2_O_3_ in glass A. As seen in Fig. 4, glass A and other borosilicate crown glasses with slight composition variations have different liquidus temperatures compared to glass B. Still, the corresponding crystal of all these glasses is the same kind of tridymite crystal. Therefore, borosilicate crown glasses have a similar glass transition process as glass B, the sodium silicate glass with 15 wt.% Na_2_O. Thus, compared to glass B, the additional boron oxide in borosilicate crown glasses does not change the formation and transformation of the embryonic clusters in these glasses. At the end of the transition, the facets of clusters in borosilicate crown glasses, which is similar to that of glass B, transform to a more ordered structure, as shown in Fig. 2. The ending temperature of this transition Tg can be experimentally determined and is expected to be close to that of glass B, which is shown in Fig. 3b to be 480 °C. Therefore, in Lebedev’s experiments, the endothermic effects of borosilicate crown glass were recorded in the temperature range between 555 and 610 °C. It is expected that slightly different temperature ranges of the endothermic effect will be observed on different specimens due to variation in experimental thermal conditions.

## Discussion and conclusion

A hundred years ago, glass research pioneers Lebedev and Tool suggested that the endothermic effects of borosilicate crown glasses between 555 and 610 °C can be attributed to its internal structural transition. However, detailed internal structures that correlate with the endothermic effects remain unknown. The recently proposed nanoflake model reveals the formation and evolution of medium-range ordering structure of glasses; and can be used to solve the century old puzzle. Based on the nanoflake model, the internal structural transition of borosilicate crown glass can be divided into two temperature regions separated by the critical temperature Tc. In the temperature region lower than Tc, the structural evolution is a one-dimensional disorder-to-order process that ends at Tg. When a glass is heated up, at temperature lower than Tg heat energy is used to increase the kinetic energy of molecules. But starting at Tg, the order–disorder transformation of glasses also requires additional heat energy, which results in the endothermic effect of glass. These marked structural changes also cause considerable modifications in the physical properties of glasses, such as its refractive index, density, and thermal expansion coefficient. The temperature value of Tg is dependent on the materials. Moreover, Tg can be different for the same material, if cooling rate during the transition is different. Recognizing these characteristics of Tg is helpful for further understanding the nature of glass and glass transitions.

It is necessary to point out that current evidence does not show whether or not the minor phase of sodium borate in the sodium borosilicate crown contributes additionally to the endothermic effect. This may present an interesting future research project. If future experiments show that sodium borate in sodium borosilicate glass indeed has an additional contribution, then the Lebedev’s results could be explained by the combination of endothermic effects produced by both phases of the glass. Since Na_2_O and B_2_O_3_ are the minor components of the borosilicate crown glass, the contribution of sodium borate to the total endothermic effect profile should be less significant than that of the silica phase. The temperature region showing strong endothermic effect should still be predominantly decided by the silica phase, and the above structural explanation for the endothermic effect of Lebedev experiments remains little changed.

## Supplementary Information

Supplementary Information 1.

Supplementary Information 2.

Supplementary Information 3.

Supplementary Information 4.

## Data Availability

The datasets used and/or analyzed during the current study available from the corresponding author on reasonable request.
