# Supplementary material for: Revisiting Lebedev’s one-century old experiment
Source: Sci Rep. 2022 Jul 31;12:13151. doi: 10.1038/s41598-022-17398-3 (PMC9339541; doi:10.1038/s41598-022-17398-3)
Supplement: Supplementary file 1 — Supplementary Information 1. [file 41598_2022_17398_MOESM1_ESM.docx]

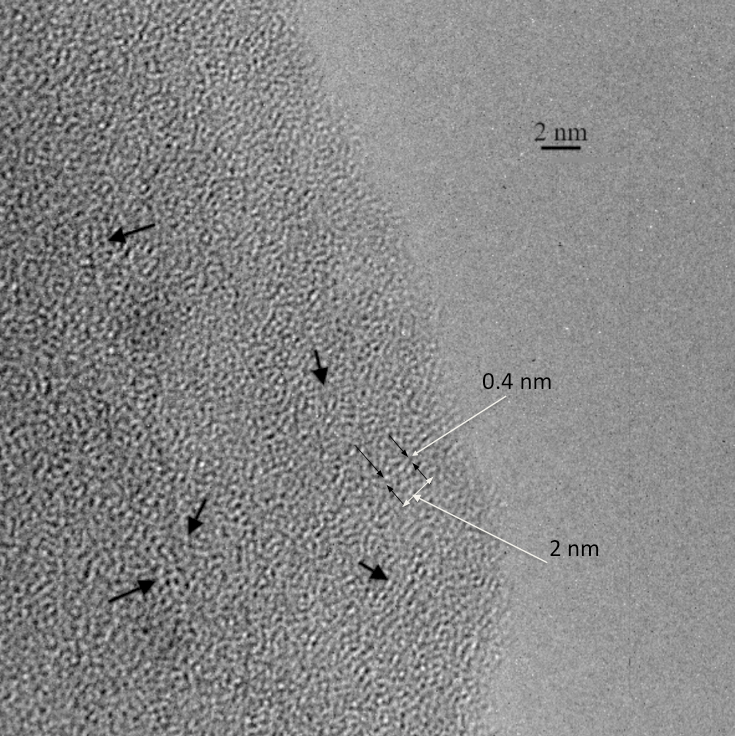


Supplementary Figure 1. TEM image of vitreous silica specimen. There are nearly parallel fringes about 1 to 2 nm long spread over the image. The spacing of these fringes is about 0.4 nm. The results confirm the two-layer structure formation in silica glass.

The figure is reproduced from a paper “A nano-flake model for the medium range structure in vitreous silica” by S. Cheng (*Phys. Chem. Glasses: Eur. J. Glass Sci. Technol. B,* April 2017, 58 (2), 33-40.**)**
