# Supplementary material for: Revisiting Lebedev’s one-century old experiment
Source: Sci Rep. 2022 Jul 31;12:13151. doi: 10.1038/s41598-022-17398-3 (PMC9339541; doi:10.1038/s41598-022-17398-3)
Supplement: Supplementary file 2 — Supplementary Information 2. [file 41598_2022_17398_MOESM2_ESM.docx]

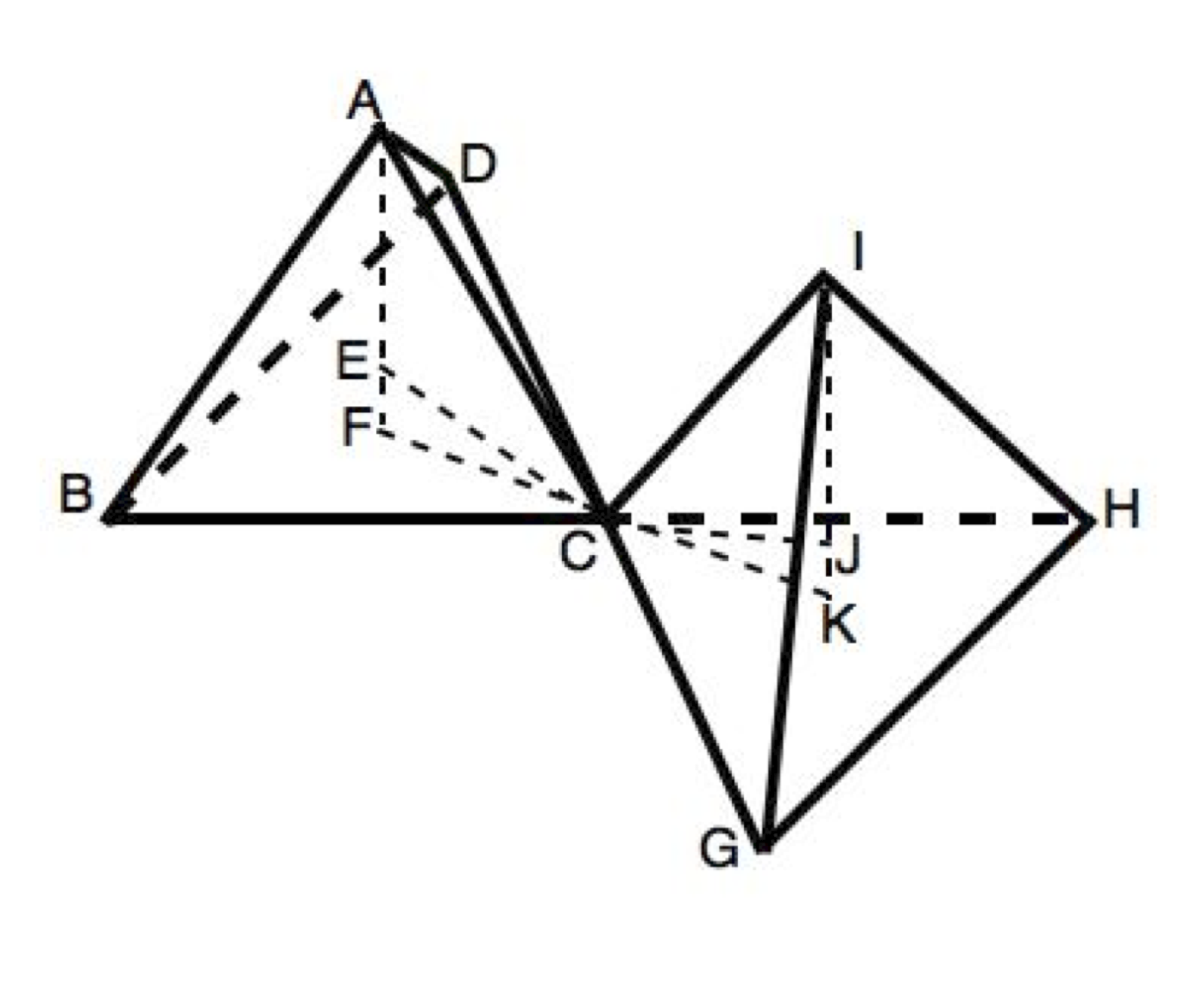


Supplementary Figure 2. Geometry and relative orientation of two corner-shared tetrahedra in a 6-membered ring of SiO_2_ tetrahedra located in a nanoflake. Point C is the position of the O atom connecting the two SiO_4_ tetrahedra, points E and J are the positions of Si atoms located in the centers of the SiO_4_ tetrahedra, and points F and K are the intersections of the extended lines AE and IJ with the planes BCD and CGH, respectively. Since points E, F, C, J, and K are located on the same plane, the Si-O-Si bond angle ∠ECJ is equal to 180º minus the angles ∠FCE and ∠JCK. Both angles ∠FCE and ∠JCK are calculated to be 19.2º; thus, the Si-O-Si bond angle is 142º. For 5- or 7-membered rings, the relative orientations of SiO_4_ tetrahedra are slightly different from that shown in the supplementary Fig. 4; the calculated Si-O-Si bond angle is 135º and 138º, respectively. Except the few O atoms located on the edges of the structure which form random Si-O-Si bond angles, the majority of O atoms are located within the layers and are not influenced by the edge effect. Since the ratio of O atoms located on the two layers to that located on the central mirror plane is 3, the most popular Si-O-Si bond angle in the structure is near 140º. The actual distribution depends on the ring distribution of the SiO_4_ tetrahedra, which is influenced by the annealing/cooling processes. Overall, the Si-O-Si bond angle distribution characteristics is very close to that obtained from X-ray scattering by Warren et al. They found that the Si-O-Si bond angle distribution is not symmetrical, ranging from 120º to 180º with a maximum at 144º. The measured most popular Si-O-Si bond angle matches that of the stabilized two-layer structure, the nanoflakes.

The two-layer structure is a one-dimensional ordering structure. As the temperature of glass increases to Tg, the order-disorder transition in glass starts. The transition requires additional heat energy, which results in the endothermic effect of glass, reported by Lebedev 100 years ago. The figure is reproduced from a paper “A nano-flake model for the medium range structure in vitreous silica” by S. Cheng (*Phys. Chem. Glasses: Eur. J. Glass Sci. Technol. B,* April 2017, 58 (2), 33-40.**)**
