# Supplementary material for: Revisiting Lebedev’s one-century old experiment
Source: Sci Rep. 2022 Jul 31;12:13151. doi: 10.1038/s41598-022-17398-3 (PMC9339541; doi:10.1038/s41598-022-17398-3)
Supplement: Supplementary file 3 — Supplementary Information 3. [file 41598_2022_17398_MOESM3_ESM.docx]

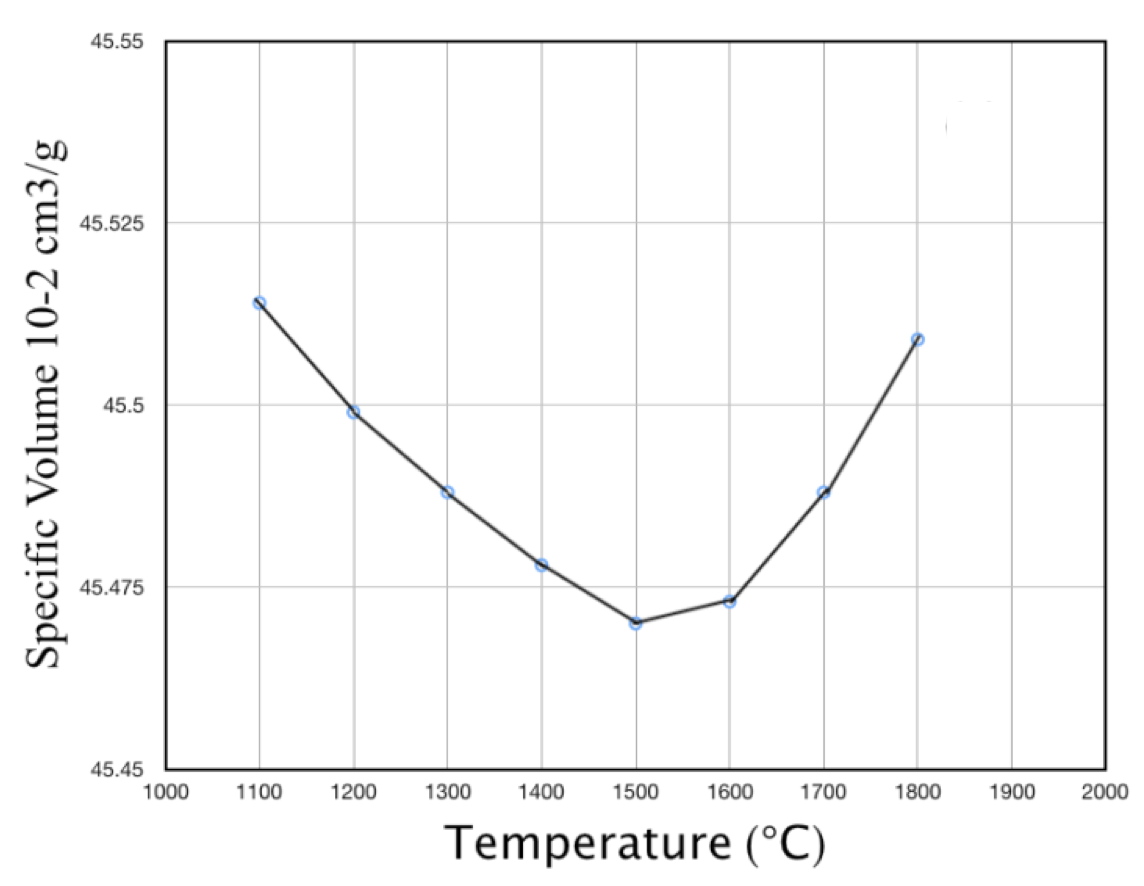


Supplementary Figure 3. The specific volume of vitreous silica as function of temperature. The graphs are produced from R. Bruckner’s experimental data in paper “Properties and structure of vitreous silica”. (*J. Non-Cryst. Solids*, **5**, 123-175, 1970)

The figures show that there is a volume minimum near 1500ºC, which is near the polymorphic inversion temperature between ß-cristobalite crystal and ß-tridymite crystal.
