# Supplementary material for: Revisiting Lebedev’s one-century old experiment
Source: Sci Rep. 2022 Jul 31;12:13151. doi: 10.1038/s41598-022-17398-3 (PMC9339541; doi:10.1038/s41598-022-17398-3)
Supplement: Supplementary file 4 — Supplementary Information 4. [file 41598_2022_17398_MOESM4_ESM.docx]

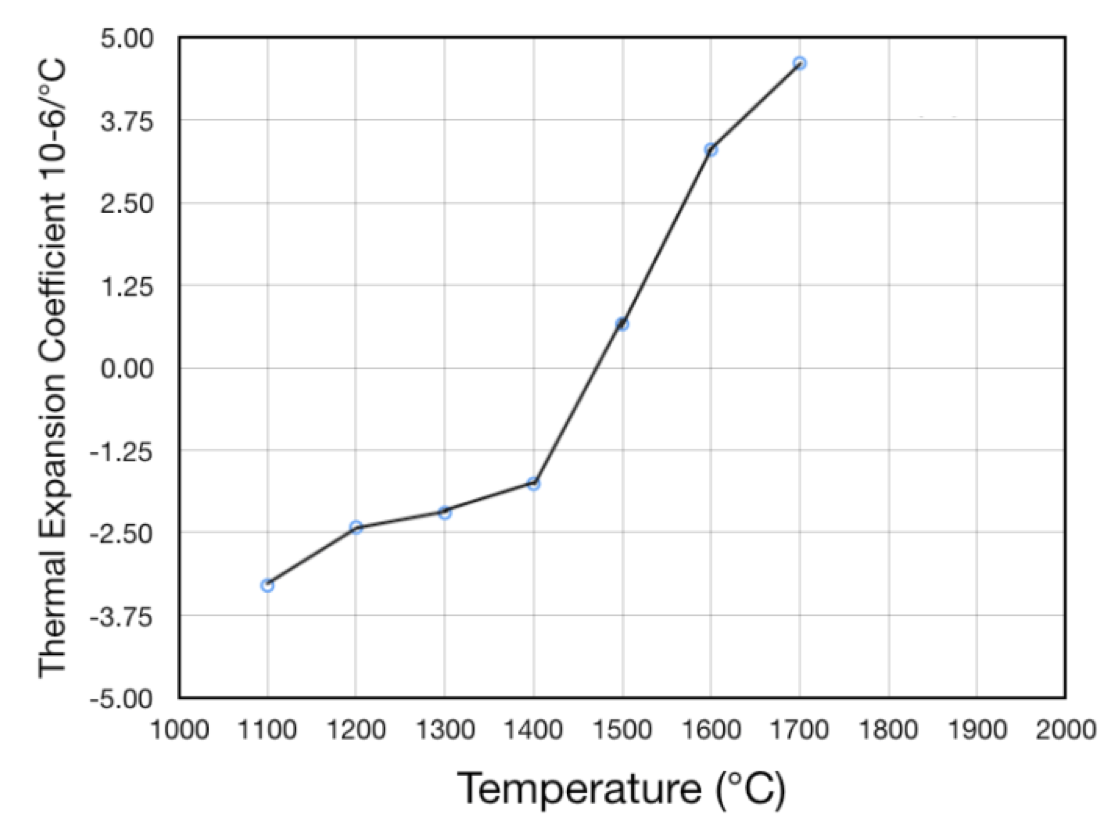


Supplementary Figure 4. The thermal expansion coefficient of vitreous silica as function of temperature. The graphs are produced from R. Bruckner’s experimental data in paper “Properties and structure of vitreous silica”. (*J. Non-Cryst. Solids*, **5**, 123-175, 1970)

The figures show that the thermal expansion coefficient exhibits a step change at around 1470ºC, the polymorphic inversion temperature between ß-cristobalite crystal and ß-tridymite crystal.
